# Serum and cutaneous transcriptional expression levels of IL31 are minimal in cutaneous T cell lymphoma variants

**DOI:** 10.1016/j.bbrep.2021.101007

**Published:** 2021-05-04

**Authors:** S. van Santen, J.J. Out, W.H. Zoutman, K.D. Quint, R. Willemze, M.H. Vermeer, C.P. Tensen

**Affiliations:** Department of Dermatology, Leiden University Medical Center, the Netherlands

**Keywords:** Cutaneous T cell lymphoma, IL31 cytokine, Pruritus

## Abstract

**Aim:**

Recent studies suggested a role for IL31 in the pathogenesis of pruritus and disease severity in patients with cutaneous T cell lymphomas (CTCL). However, discrepant results were reported for IL31 serum levels, transcriptional expression levels or immunohistochemistry studies and its relation to pruritus intensity and/or disease severity in CTCL. Most studies did not distinguish between different CTCL variants. We investigated IL31 serum levels in different subtypes of CTCL, including Mycosis Fungoides (MF) (typically not pruritic), Folliculotropic Mycosis Fungoides (FMF) and Sézary syndrome (SS) (both often pruritic).

**Methods:**

From 54 CTCL patients (17 SS, 21 FMF and 16 classic MF) serum samples were analyzed with a high sensitivity V-PLEX immunoassay for IL31. The study group included 35/54 (65%) patients with complaints of pruritus. Thirty-five patients had advanced stage disease (≥stage IIB). A visual analog scale score (VAS score) for pruritus was available in 29 CTCL patients (7 SS, 9 FMF and 13 classic MF) and in other cases complaints of pruritus were retrieved from medical records. qPCR analyses for *IL31* expression were performed in lesional skin biopsies from 8 CTCL patients. Serum samples from 4 healthy individuals without pruritus and from 5 atopic dermatitis (AD) patients with severe pruritus were included as controls.

**Results:**

In 11/54 (20%) of CTCL patients low serum levels of IL31 were detected (mean 0.48 pg/mL, range 0.20–1.39 pg/mL) including 6/17 (35%) SS patients (mean 0.57 pg/mL) and 5/21 (24%) FMF patients (mean 0.33 pg/mL). All 11 patients with detectable levels of IL31 reported complaints of moderate to severe pruritus and 9/11 patients presented with advanced stage disease (≥IIB). qPCR analyses resulted in lowly expressed *IL31* expression levels in 4 of 8 patients; these patients all suffered from pruritus and advanced stage disease.

**Conclusions:**

Translational and transcriptional expression levels of IL31 were very low or undetectable in CTCL patients. Detectable low IL31 serum levels were exclusively observed in SS and FMF patients and not in patients with classic MF. However, these marginal IL31 levels in a small proportion of CTCL patients do not support an essential role for IL31 in CTCL patients.

## Highlights

• Interleukin 31 (IL31) is suggested to play a key role in pruritus.
• Cutaneous T cell lymphoma (CTCL) patients may suffer from severe pruritus.
• Previous reports show variable results regarding the role of IL31 in CTCL patients.
• Minimal to undetectable IL31 serum levels were found in 54 CTCL patients.
• These observations do not support a prominent role for IL31 in CTCL.

## List of abbreviations

AD: Atopic Dermatitis
CD45RO: Cluster of Differentiation 45RO (marker for activated T cell)
cDNA: complementary DNA
CLA: Cutaneous Lymphocyte Antigen
CTCL: Cutaneous T Cell Lymphomas
FMF: Folliculotropic Mycosis Fungoides
IL31: Interleukin 31
IL31RA: Interleukin 31 Receptor A
MF: Mycosis Fungoides
OSMR: Oncostatin M Receptor
qPCR: quantitative Polymerase Chain reaction
SS: Sézary Syndrome
Th2: T-helper 2
TNM: Tumor-Node-Metastasis
VAS: Visual Analog Scale

## 1 Introduction

Cutaneous T cell lymphomas (CTCL) are a heterogeneous group of malignant non-Hodgkin lymphomas of skin-homing T-lymphocytes, of which Mycosis fungoides (MF) and Sézary syndrome (SS) are the most commonly investigated variants [1]. Pruritus is a common sign in CTCL patients which can be a therapeutically challenging to manage [2]. The underlying mechanisms causing pruritus in these patients are currently poorly understood. Recent studies found pruritic skin inflammation in transgenic mice overexpressing IL31 and demonstrated significantly elevated serum and mRNA IL31 levels in atopic dermatitis (AD) and prurigo nodularis suggesting a role for IL31 in the pathogenesis of pruritic skin diseases [[3], [4], [5], [6], [7]]. Although most studies predominantly focused on patients with atopic dermatitis (AD), a few studies investigated IL31 in CTCL patients as well [[8], [9], [10], [11], [12]]. IL31 is a cytokine that is predominantly produced by activated T-helper 2 (Th2) cells and CLA + CD45RO + effector memory T cells and signals through a heterodimeric receptor composed of IL31 receptor A (IL31RA) and oncostatin M receptor (OSMR) [3]. Recent findings with IL31RA antigen therapy showed a reduction of pruritus in AD patients [13], indicating that IL31 might be of similarly therapeutic interest in CTCL patients. However, previous studies focusing on IL31 in CTCL patients have reported variable results regarding the correlation between IL31 levels and pruritus intensity [9,10,12], disease severity [8,11] or both parameters [8]. However, these studies typically did not distinguish between different CTCL variants. Because Sézary syndrome (SS) and the folliculotropic variant of Mycosis Fungoides (FMF) are CTCL variants that are characterized by (intense) pruritus while classic Mycosis Fungoides (MF) is typically not, we hypothesized that differences in pruritic and non-pruritic variants of CTCL might explain discrepancies reported in literature and that pruritic CTCL patients may be associated with higher levels of IL31. The aim of the current study was to investigate translational IL31 levels in serum and cutaneous *IL31* transcriptional expression in pruritic and non-pruritic CTCL variants and to study the relation of IL31 with regard to pruritus and clinical disease stage.

## 2 Material and methods

### 2.1 Patients, samples and controls

From 54 CTCL patients (16 classic MF, 21 FMF and 17 SS) a total of 68 serum samples (16 classic MF, 25 FMF, 27 SS) were included in the study (Table 1, Table 2). In all cases the diagnosis had been established by an expert panel of dermatologists and pathologists, highly experienced in CTCL and in accordance with the WHO-EORTC classification [1]. Staging at time of sample collection was performed according to the Tumor-Node-Metastasis (TNM) classification for MF and SS [14] and resulted in 19 early-stage (stage I-IIA) and 35 advanced-stage (stage IIB-IV) CTCL patients. From ten patients, two or three consecutive serum samples had been included in this study. These samples had been obtained at different time points during a patients' disease course while patients remained to have either early- or advanced-stage disease. Information whether a patient did or did not suffer from pruritus at time of sampling had been retrieved from medical records or from a visual analog scale score (VAS score) for pruritus which had been available in 29 CTCL patients (13 classic MF, 9 FMF and 7 SS). Patients with an available VAS score were considered to suffer from pruritus when VAS was ≥3.3 (VAS ≥3.3–6.6 moderate pruritus; VAS ≥6.6 severe pruritus). Patients with VAS scores <3.3 were considered to have minimal pruritus and were not counted as patients suffering from pruritus. Serum samples had been stored at −80 °C until use. Thirty-one of 68 serum samples had been collected over a 5 month-period prior to IL31 analysis and the remaining 37 derived from our biobank and had been stored for a median duration of 6 years (range 1–16 years) until IL31 analysis. Serum of 4 healthy individuals and 5 AD patients with severe pruritus (VAS scores ranging from 6.7 to 8.7) were included as controls for IL31 determination. The study was performed in accordance with our in-house biobank protocol (B20.046), approved by the institutional review board committee of Leiden University Medical Center, the Netherlands and in accordance with the principles of the Declaration of Helsinki. Patients’ written informed consent was obtained prior to collection of blood serum.

**Table 1:** Patient characteristics and IL31 levels.^a^.

Diagnosis	No. Patients	Stage	Stage	Pruritus	No pruritus	Available VAS score	No. Patients with detectable IL31 (mean, pg/mL)	
		I-IIA	IIB-IVB					
**SS**	17	–	17	16	1	7	6 (0.57)	
**FMF**	21	9	12^b^	16	5	9	5 (0.33)	
**MF**	16	10	6^c^	3	13	13	0 (-)	
**Total**	**54**	**19**	**35**	**35**	**19**	**29**	**11 (0.48)**	

SS Sézary syndrome, FMF folliculotropic mycosis fungoides, MF classic mycosis fungoides, No. number, I-IIA early stage of disease according to the International Society of Cutaneous Lymphomas (ISCL) [14], IIB-IVB advanced stage of disease according to ISCL, VAS visual analog scale (for pruritus).

a See detailed information on this study cohort in Table 2.

b 10 out of 12 patients with advanced stage FMF reported moderate to severe pruritus.

c 1 out of 6 patients with advanced stage MF reported moderate to severe pruritus.

**Table 2:**
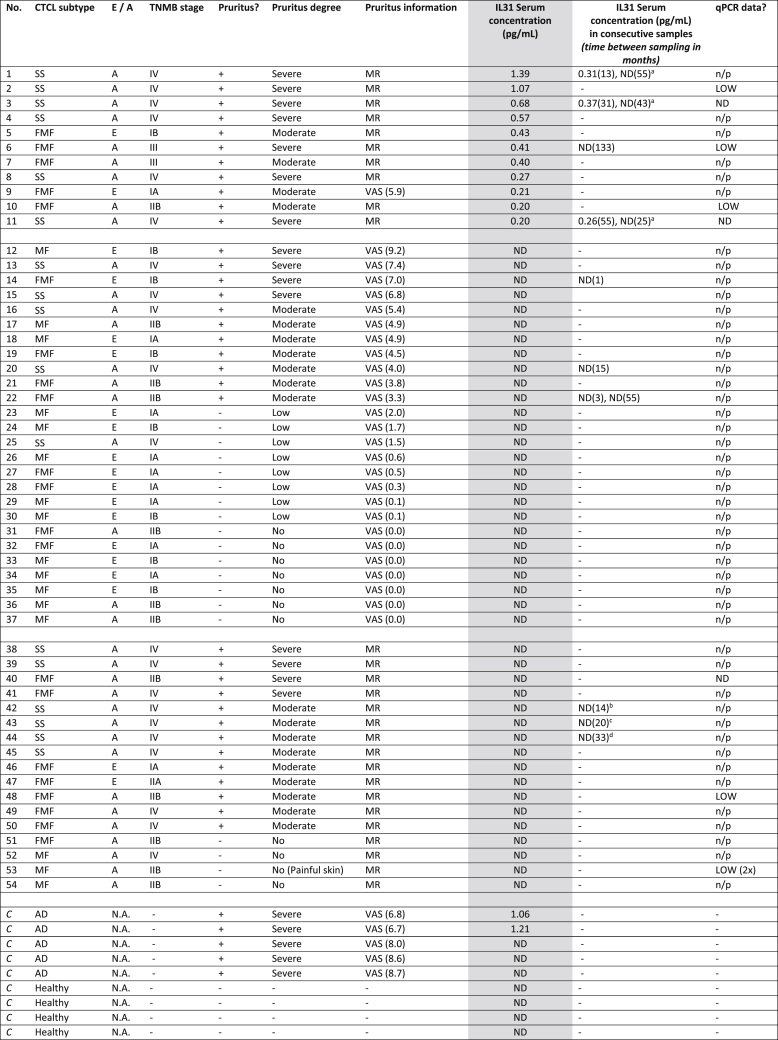
Detailed characteristics of included CTCL patients and controls, IL31 serum detectability and relation with pruritus.fx1

A: advanced-stage disease (according to TNMB staging system stages IIB-IV are considered advanced-stage disease); AD: atopic dermatitis; CTCL: cutaneous T cell lymphoma; C: control sample; E: early-stage disease (stages IA-IIA are considered early-stage disease); FMF: folliculotropic mycosis fungoides; mo: months; LOW: lowly expressed (Cq > 31); MF: mycosis fungoides, MR: (retrieved from) medical records; N.A. not applicable; ND: not detected; No.: patient number; n/p: not performed; qPCR quantitative polymerase chain reaction; SS: Sézary syndrome; TNMB: tumor node metastasis blood staging system; VAS: visual analog scale (VAS <3,3 was considered low; VAS 3,3–6,6 was considered moderate, VAS >6,6 was considered severe).

^a^ In these patients IL31 could be detected in one of two follow-up samples, while these patients continued to suffer from a similar degree of pruritus.

^b^ At follow up sampling, this SS patient had no complaints of pruritus (VAS score of 0.0).

^c^ At follow up sampling, this SS patient suffered from minimal complaints of pruritus (VAS score of 2.0).

^d^ At follow up sampling, this SS patient had no complaints of pruritus (retrieved from medical record).

### 2.2 IL31 immunoassay

**Figure 1:**
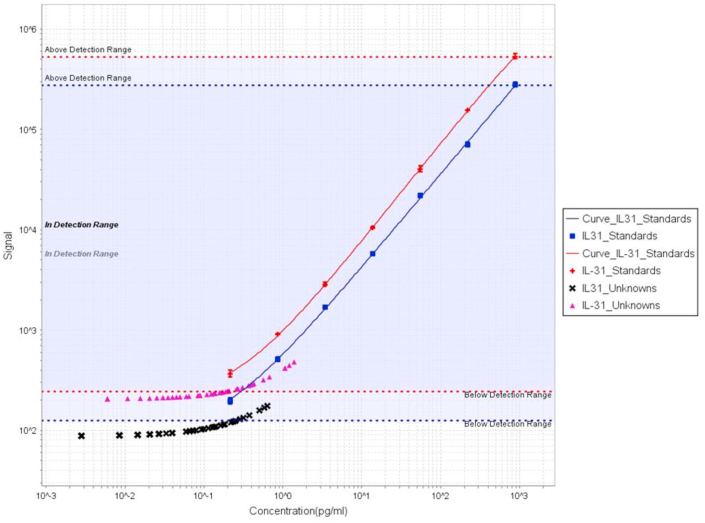
Standard curves for IL31 V-PLEX multispot analysis (Th17 panel) and single analyte analysis. Standard curves for both the original experiment and the verification experiment demonstrate similar observations and reveal that a minority of CTCL serum samples exceed the IL31 detection threshold.

IL31 concentrations in serum samples from different CTCL patient groups and controls were examined using a sensitive V-PLEX multi-spot assay (Th17 panel 1 (human), K15085D, MSD Maryland, USA). Verification of our results was done with the IL31, SECTOR K151XAD-1 (MSD) (IL31 single analyte assay). As a quality control, a standard curve with 8 dilutional steps was run on the same plate along with the investigated CTCL serum samples for both the multi-spot V-PLEX assay as for the verification single analyte assay (Fig. 1).

### 2.3 qPCR analysis

Quantitative PCR (qPCR) to determine IL31 mRNA expression was performed in 8 CTCL patients of which frozen skin biopsies (n = 9, 2 biopsies originated from the same patient) were obtained at same date of serum sample collection. RNA was isolated using the RNeasy Mini Kit (Qiagen, Hilden, Germany), which included on-column DNase digestion. cDNA synthesis was performed using the iScript cDNA Synthesis Kit (Bio-Rad, Veenendaal, The Netherlands) and qPCR was performed with SYBR Green Super mix (Bio-Rad). *IL31* expression was examined using qPCR on a CFX384 PCR detection system (Bio-Rad) by measuring relative gene expression against stably expressed reference genes *ERCC3* and *TMEM87A* [15] using intron-spanning primers (Supplementary Table 1) according to the delta-delta Cq method [16].

## 3 Results

### 3.1 Serum levels

Results are displayed in Table 1 (summarized) and Table 2. In 11/54 (20%) CTCL patients serum levels of IL31 could be detected (mean 0.48 pg/mL, range 0.20–1.39 pg/mL). These included 6/17 (35%) SS patients (mean 0.57 pg/mL, range 0.20–1.39 pg/mL) and 5/21 (24%) FMF patients (mean 0.33 pg/mL, range 0.20–0.43 pg/mL); IL31 could not be detected in classic MF. Nine of 11 patients with detectable levels of IL31 presented with advanced stage disease (≥IIB) and reported complaints of (moderate to severe) pruritus. Still, in 69% of patients that had reported (moderate to severe) pruritus IL31 could not be detected. Information on pruritus in our 11 detectable cases had been available from medical records in 10 cases and in only 1 case a VAS score was available (score 5.9). As a consequence, correlation analysis between VAS scores and IL31 concentrations could not be performed. In three patients with consecutive serum samples at various time points, IL31 could be detected repeatedly in low amounts (in 2 of 3 included samples). In the other patients with consecutive serum samples, IL31 could not be detected. Serum levels of IL31 could be detected in two out of five patients with AD (1.06 and 1.21 pg/mL with VAS scores of 6.8 and 6.7 respectively) and were not detectable in four healthy controls. Since IL31 is one of the eight measured cytokines in the utilized Th17 panel (including IL17A, IL21, IL22, IL23, IL27, IL31, and MIP3α) we have been able to compare cytokine levels relative to IL31. We observed that among the total set of measurable cytokines, IL31 was ubiquitously lowly expressed or undetectable. Positive as well as negative results from both patient and control samples were confirmed using a high-sensitivity single analyte IL31 assay.

### 3.2 qPCR

In 4 of 8 patients *IL31* could be detected in very low, not in quantitatively measurable levels (Cq > 31) (Supplementary Fig. 1). These four patients suffered from pruritus (one case described a painful skin) and advanced stage disease and in two patients very low IL31 serum levels were detected.

## 4 Discussion

Pruritus is a common distressing symptom in CTCL patients that may be challenging to manage therapeutically [17]. Over the past years, IL31 has been reported to play a central role in the pathogenesis of pruritus [3,4] and an IL31 receptor antagonist is being developed as a novel therapeutic strategy for the reduction of pruritus in AD patients [13].

The aim of the current study was to investigate the role of IL31 in CTCL patients. This study was the first investigating the role of IL31 among the following three (major) CTCL subtypes: classic MF, FMF and SS. It is known that pruritus in CTCL is more common in FMF and SS compared to classic MF and is more prevalent among patients with advanced stage disease [2,18]. Therefore, at the start of our study, we hypothesized that serum samples from the more pruritic variants of CTCL (FMF and SS) and from more advanced stages of disease express differentially higher IL31 serum levels. The results of our study revealed very low IL31 serum levels in 11 CTCL patients but undetectable levels in the remaining 43 CTCL patients. As a matter of fact, IL31 was exclusively found in serum from FMF and SS patients, was not detectable in patients with classic MF and concerned advanced stages of disease in 82% of cases. It can therefore not be excluded that marginal concentrations might play a role in at least a minority of CTCL patients suffering from pruritus or advanced stage disease. However, in 69% of all patients that reported moderate to severe pruritus in our cohort, IL31 could not be detected, questioning an important role for IL31 related pruritus in our CTCL patients. In literature, s tudies focusing on IL31 in serum of CTCL patients have shown diverse findings. O ur results are in line with the study of Möbs et al. that reported equally low IL31 serum values and only few samples exceeded the threshold for unequivocal quantification [12]. Several other studies reported increased IL31 serum concentrations in CTCL [8,9,11], but in all these studies the concentration of IL31 was low when compared to values reported in AD patients [5,6,19]. Likewise, in the study of Singer et al. not all CTCL patients demonstrated detectable IL31 serum values while only leukemic stages of disease were investigated [9]. Being aware of the previously published low IL31 concentrations in CTCL, for the current study we selected a highly sensitive immunoassay (V-PLEX multi-spot and single analyte) characterized by a large dynamic range. In addition, the serum diluents used in these immunoassays have been optimized to eliminate possible interferences from heterophilic antibodies, a known source of non-specific signals in ELISA based assays [20]. With respect to disease severity, our results found almost all detectable IL31 serum levels and transcriptional levels in advanced stages of disease, which is in line with the study of Ohmatsu et al. that found a correlation between IL31 concentrations and CTCL disease severity [8]. However, it should be noted that it is difficult to determine whether our observation is biased by the relative small proportion of positive samples; as other cohorts with few positive samples found no relation with disease stage [12]. As we found that in the large majority of CTCL patients with moderate to severe pruritus IL31 could not be detected in serum or skin, this might suggest that other mediators are more important in the pathogenesis of pruritus in CTCL, as has been proposed before [11]. Although some studies found a relation between the amount of IL31 and pruritus severity in immunohistochemistry studies [10], other studies reported that cutaneous IL31 in human subjects may not exert a direct pruritic effect in the skin, thus questioning a direct relation between cutaneous IL31 and pruritic symptoms [21]. In line with our findings in serum, we found that transcriptional expression for *IL31* in skin was not quantifiable further suggesting that alternative mechanisms are more important in the pathogenesis of pruritus in CTCL.

## 5 Conclusions

In conclusion, using a highly sensitive V-PLEX IL31 immunoassay on well characterized patient groups, we demonstrated very low IL31 serum levels in a minority of patients with pruritic CTCL variants (FMF and SS) and no detectable levels in classic MF patients. Expression of IL31 mRNA in pruritic CTCL skin was similarly low and could not be detected in quantitative measurable levels, by means of qPCR. We cannot exclude that marginal concentrations might play a role in at least a minority of CTCL patients suffering from pruritus or advanced stage disease. However, our observations do not support a prominent role for IL31 in the pathogenesis of pruritus and disease activity in CTCL patients and further studies elucidating crucial mechanisms in CTCL pruritus are highly warranted.

## Funding sources

This research did not receive any specific grant from funding agencies in the public, commercial, or not-for-profit sectors.

## Author contributions

S van Santen: Data curation, Formal Analysis, Project administration, Writing - Original Draft preparation, VisualizationJJ Out: Formal analysis, ValidationW Zoutman: Formal analysis, ValidationKD Quint: Writing - Review & EditingR Willemze: Writing - Review & EditingMH Vermeer: Conceptualization, Supervision, Writing - Review & EditingCP Tensen: Conceptualization, Methodology, Supervision, Writing - Review & Editing.

## Declaration of competing interest

None.

## Figures and Tables

**Figure:**
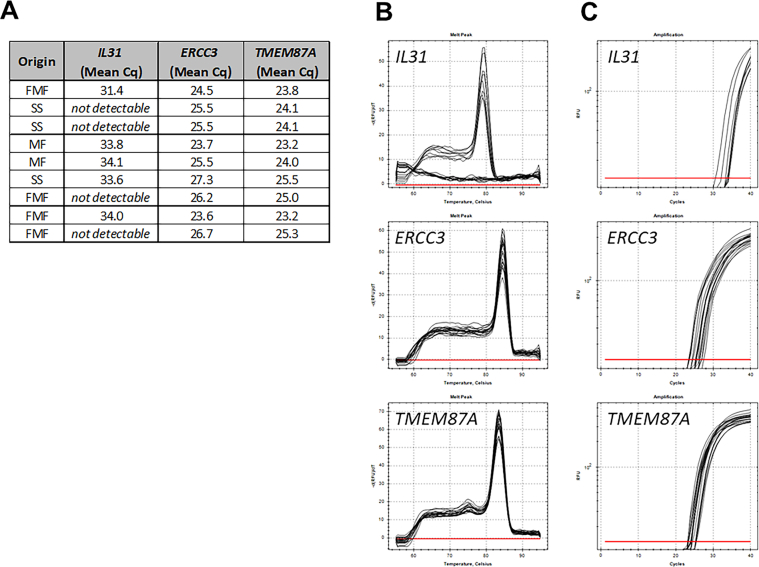
Supplementary Figure-1 *IL31* qPCR results from 9 CTCL skin biopsies. (A) Mean c ycle quantification (Cq) values for *IL31* and reference genes *ERCC3* and *TMEM87A* as performed in duplicate. (B) Post-PCR assessment of amplification specificity by melting curve analysis. (C) Log-transformed amplification plots. *NB: results from patient with MF belong to the same patient (see also patient 53 from*Table 2*). FMF: folliculotropic mycosis fungoides, SS: Sézary syndrome, MF: classic mycosis fungoides.*
