# Supplementary material for: Serum and cutaneous transcriptional expression levels of IL31 are minimal in cutaneous T cell lymphoma variants
Source: Biochem Biophys Rep. 2021 May 4;26:101007. doi: 10.1016/j.bbrep.2021.101007 (PMC8121649; doi:10.1016/j.bbrep.2021.101007)
Supplement: Multimedia component 1 [file mmc1.docx]

**Supplementary Table 1. Primer sequences for qPCR**
